# Obestatin stimulates glucose-induced insulin secretion through ghrelin receptor GHS-R

**DOI:** 10.1038/s41598-017-00888-0

**Published:** 2017-04-20

**Authors:** Geetali Pradhan, Chia-Shan Wu, Jong Han Lee, Preeti Kanikarla, Shaodong Guo, Vijay K. Yechoor, Susan L. Samson, Yuxiang Sun

**Affiliations:** 1 USDA/ARS Children’s Nutrition Research Center, Department of Pediatrics, Baylor College of Medicine, Houston, TX USA; 2 Interdepartmental Program in Translational Biology and Molecular Medicine, Baylor College of Medicine, Houston, TX USA; 3 Department of Nutrition and Food Science, Texas A&M University, College Station, TX USA; 4 College of Pharmacy, Gachon University, Incheon, 21936 Korea; 5 Department of Medicine, Baylor College of Medicine, Houston, TX USA

## Abstract

Orexigenic hormone ghrelin and anorexic hormone obestatin are encoded by the same preproghrelin gene. While it is known that ghrelin inhibits glucose-stimulated insulin secretion (GSIS), the effect of obestatin on GSIS is unclear. Ghrelin’s effect is mediated by its receptor Growth Hormone Secretagogue Receptor (GHS-R), but the physiologically relevant receptor of obestatin remains debatable. Here we have investigated the effect of obestatin on GSIS *in vitro*, *in vivo* and *ex vivo*, and tested whether obestatin regulates insulin secretion through GHS-R. We found that under hyperglycemic condition, obestatin augments GSIS in rat insulinoma cells (INS-1) and in pancreatic islets from *ghrelin* ^−/−^ mice. Surprisingly, obestatin-induced GSIS was absent in β-cells in which GHS-R was suppressed. Obestatin-induced insulin secretion was abolished in the circulation of *Ghsr* ^−/−^ mice, and in pancreatic islets isolated from *Ghsr* ^−/−^ mice. We also found that obestatin-induced GSIS was attenuated in islets isolated from β-cell-specific *Ghsr* knockout MIP-Cre/ERT;Ghsr^f/f^ mice. Our data collectively demonstrate that obestatin is a potent insulin secretagogue under hyperglycemic condition, and obestatin’s effect on insulin secretion is mediated by GHS-R in pancreatic β-cells. Our findings reveal an intriguing insight that obestatin and ghrelin have opposing effects on insulin secretion, and both are mediated through ghrelin receptor GHS-R.

## Introduction

Type 2 diabetes mellitus (T2DM) is a complex multifactorial disease that is characterized by insulin resistance, loss of pancreatic β-cell mass, and diminished β-cell function. Impairment of β-cell glucose-stimulated insulin secretion (GSIS) is a hallmark of type 2 diabetes. Understanding the regulation of insulin secretion in β-cells is essential for developing therapies for T2DM. Obestatin is a 23-amino-acid anorexic hormone that has been found to exert favorable effects on glucose homeostasis by increasing β cell mass, and by reducing systemic insulin resistance and adipose tissue inflammation^1–5^. These beneficial effects of obestatin make it an attractive therapeutic candidate for prevention/treatment of T2DM.

Anorexic hormone obestatin is encoded by the preproghrelin gene that also encodes the orexigenic hormone ghrelin^6^. While high levels of obestatin are expressed in the oxyntic mucosa of the stomach, lower levels of obestatin are also expressed in pancreatic islets^1, 7, 8^ and some other tissues^2, 9–11^. In the pancreas, obestatin is expressed in ε-cells of pancreatic islets, while ghrelin is expressed in α, β and ε-cells of the pancreatic islets^1, 12–16^. It is known that GHS-R is expressed in α, β and δ-cells of the pancreatic islets^4, 17–21^. Obestatin has been shown to antagonize ghrelin’s effects on appetite, food intake, gastric emptying^6, 22^, as well as growth hormone secretion^23, 24^. Obestatin is reduced in obese humans^8^. It has been reported that obestatin is positively correlated with insulin sensitivity^25^, and obestatin protects against diet-induced insulin resistance^2^. More interestingly, it has been shown that obestatin increases β-cell proliferation and survival, and reduces inflammation-induced β-cell apoptosis^1^. Furthermore, obestatin has been shown to protect against streptozotocin (STZ)-induced β-cell failure in rats^26^.

We and others have reported that ghrelin inhibits insulin secretion^4, 27–31^, but the effect of obestatin on insulin secretion is not clear. While some studies showed that obestatin increases insulin secretion in human islets and diet-induced obese mouse islets^1^, others showed that obestatin inhibits insulin secretion^7, 32^ or has no effect on insulin secretion^33–35^. To date, GHS-R is the only identified physiologically relevant receptor for ghrelin; ghrelin is the only ligand that is confirmed to bind to and function through GHS-R. Since the discovery of obestatin, the receptor of obestatin has been controversial. Initially, obestatin was suggested to be the endogenous ligand of orphan G-protein-coupled receptor 39 (GPR39)^6, 36^. However, subsequent studies failed to confirm specific binding of obestatin to GPR39^37–40^. Recently, some studies suggested that obestatin regulates survival and proliferation of pancreatic β-cells via glucagon-like peptide-1 receptor (GLP-1R)^1, 4^, but others failed to detect the direct binding of obestatin to GLP-1R^35^. Interestingly, evidence does suggest that obestatin binds to a G-protein-coupled receptor (GPC-R)^41–44^, and GHS-R is a heterotrimeric GPC-R^40^. Further, it has been shown that obestatin interacts with ghrelin binding sites, and pharmacological blocking of GHS-R abolishes obestatin’s protective effects on cell survival^1^.

Our study aims to investigate the effect of obestatin on GSIS and determine whether obestatin’s effect on GSIS is mediated through GHS-R. First, to study the effects of obestatin on insulin secretion, we utilized rat insulinoma INS-1 cells, as well as mouse pancreatic islets from *ghrelin* ^−/−^ mice lacking both ghrelin and obestatin. Next, to investigate whether GHS-R mediates obestatin-induced GSIS, we used various model systems: a) *in vitro* system - INS-1 cells with either GHS-R pharmacologically blocked by GHS-R antagonists YIL781 & JMV2959, or *Ghsr* transiently suppressed by siRNA. b) *in vivo* system - Mice with *Ghsr* deleted globally (*Ghsr* ^−/−^). c) *ex vivo* – Isolated islets from *Ghsr* ^−/−^ mice and β-cell-specific *Ghsr* knockout mice (MIP-Cre/ERT;Ghsr^f/f^). We demonstrated that obestatin augments insulin secretion in pancreatic β-cells under hyperglycemic condition, and obestatin-induced stimulatory effect on insulin is mediated through GHS-R. To our knowledge, this is the first set of comprehensive evidences showing that obestatin’s effect on GSIS is mediated through ghrelin receptor GHS-R.

## Research Design and Methods

### Animals

All experiments were approved by the Animal Care Research Committee of the Baylor College of Medicine. Animals were housed under controlled temperature and lighting (75 ± 1 °F; 12 h light-dark cycle) with free access to regular mouse chow and water. Our *ghrelin* ^−/−^, *Ghsr* ^−/−^ mice on C57BL/6 J background were generated and characterized as we have previously described^18, 45^. Briefly, *ghrelin* ^−/﻿−^ mice were backcrossed to C57BL/6 J background for 10 generations and *Ghsr* ^−/−^ mice were backcrossed for 15 generations. All mice used in the experiments were age-matched adult congenic males. WT and homozygous knockout mice (*ghrelin* ^−/−^, *Ghsr* ^−/−^) were housed and bred in a pathogen-free facility at Baylor College of Medicine, and all methods were performed in accordance with the relevant guidelines and regulations.

*Ghsr* ^*f*/*f*^ mice were originally obtained from Taconic Farms and were backcrossed as recently reported^46^, and then bred with MIP-Cre/ERT^47^ mice to generate MIP-Cre/ERT;Ghsr^*f*/*f*^. The *Ghsr* ^*f*/*f*^ and MIP-Cre/ERT;Ghsr^*f*/*f*^ mice were gavaged five times with tamoxifen (4 mg/200 ul) in peanut oil on alternate days.

### Reagents

Rat/Mouse obestatin peptide was from Phoenix Pharmaceuticals (Burlingame, CA). The GHS-R antagonists used were YIL781 (Tocris Bioscience, Bristol, UK) and JMV2959 (Calbiochem, San Diego, CA).

### Cell Culture and siRNA transfection

INS-1 cells (832/13 cells) were a generous gift from Chris Newgard (Duke University)^48^. The cells were cultured in RPMI-1640 (Corning Incorporated, NY) containing 11.1 mM D-glucose supplemented with 10% inactivated fetal bovine serum, 100 U/ml penicillin, 100 µg/ml streptomycin, 10 mM HEPES, 2 mM L-glutamine, 1 mM sodium pyruvate and 50 µM β-mercaptoethanol^48^.

For siRNA knockdown of *Ghsr* expression, INS-1 cells were plated in a 24-well plate at 0.5*10^−6^ cells/well and cultured until confluent; they were then transfected with scramble/si*Ghsr* and Lipofectamine 2000 (Invitrogen, Carlsbad, CA) as per the manufacturer’s instruction_._ Briefly, 2.5 μl of 40 μM scramble/si*Ghsr* was added to 60 μl Opti-MEM media (Invitrogen, Carlsbad, CA) in an RNase-free tube. In a separate RNase-free tube, 2.5 μl Lipofectamine2000 (Invitrogen, Carlsbad, CA) was added to 60 μl Opti-MEM media, mixed and incubated for 5 min at room temperature. After 5 min, the scramble/si*Ghsr* mix was combined with the Lipofectamine2000 mix to a total volume of 125 μl. The solution was mixed gently and incubated at room temperature for 20 mins. During the incubation, the RPMI-1640 medium in the 24-well plate was replaced with 125 ul low serum RPMI-1640 media containing 5% FBS and 1% INS-1 supplement without any antibiotic. After 20 min, the scramble/siRNA/Lipofectamine2000 mix was added to the wells. The final siRNA concentration is 0.4 uM in a final volume of 250 ul/well. The plate was then incubated for 4 hours at 37 °C in a humidified incubator with 5% CO_2_. After 4 hours, 1 ml of RPMI-1640 was added to each well and plate was incubated at 37 °C in the incubator. After overnight culture, the media was replaced with 1 ml of fresh RPMI-1640 media and incubated overnight. 48 hours after the start of transfection, the cells were harvested and used for insulin secretion or RNA/protein analysis. The siRNA sequences were designed with ABI software and synthesized by Sigma-Aldrich Scramble (Sense strand): 5′-CAACAACGAAGCGACAUAAUC-3′; Scramble (Antisense strand): 5′-UUAUGUCGCUUCGUUGUUGUC-3′ or si*Ghsr* (Sense strand) 5′-CCACAAACAGACAGUGAAGUU-3′; si*Ghsr* (Antisense strand): 5′-CUUCACUGUCUGUUUGUGGUU-3′.

### Insulin secretion experiments

INS-1 cells (passage numbers 24–40) were plated in a 24-well plate at a density of ~0.5 × 10^−6^ cells/well and were grown to near confluence. For islet culture experiments, islets were collected using the collagenase method (Collagenase P, Roche, Basel, Switzerland)^49^ and were cultured overnight. Briefly, 3 ml of 1 mg/ml collagenase P was injected into the opening of the pancreatic duct using a 27 G needle. Pancreas was then removed and placed in 50 ml tube containing 2 ml of the collagenase solution. The tube was later placed in 37 °C water bath for 12–13 minutes with 100–120 shakings/min until the tissues were homogenously digested. 20 ml cold HBSS was subsequently added to stop the digestion and the tubes were centrifuged at 290 g for 30 seconds at 4 °C. The islets were then centrifuged using a Ficoll gradient Histopaque-1077 (Sigma-Aldrich, Kawasaki, Japan) per manufacturer’s instruction. Lastly the islets were handpicked and incubated overnight in RPMI-1640 medium containing 10% fetal bovine serum, 100 U/ml penicillin, 100 µg/ml streptomycin, 10 mM Hepes, 2 mM L-glutamine, 1 mM Sodium-pyruvate, 0.05 mM 2-mercaptoethanol and 5.5 mM glucose. The next day, the medium was replaced with Hanks’ Balanced Salt solution (HBSS) pH 7.2, consisting of 114 mM NaCl, 4.7 mM KCl, 1.2 mM KH2PO_4_, 1.16 mM MgSO_4_, 20 mM HEPES, 2.5 mM CaCl_2_, 25.5 mM NaHCO^3^ 0.2% bovine serum albumin and 3.3 mM glucose for 2 hr^48^. For insulin secretion experiments, cells were incubated in 0.5 ml of the secretion media mentioned above, but containing the indicated glucose concentrations with or without obestatin. For GHS-R antagonist experiments, cells were incubated with 5 µM of antagonist in the HBSS buffer, containing 3.3 mM glucose for 1 hour prior to obestatin treatment; the buffer was then replaced with 500 µl of fresh HBSS containing high glucose (22.2 mM) with the indicated concentrations of obestatin and/or antagonist.

### Real-time RT-PCR

Total RNA from islets was isolated using Arcturus PicoPure RNA isolation kit (ABI) following the manufacturer’s instructions. cDNA was synthesized from 250–500 ng RNA using the SuperScript III First-Strand Synthesis System (Invitrogen, Carlsbad, CA). Real-time RT-PCR was performed on Bio-Rad real time PCR cycler (Bio-Rad Lab., Hercules, CA) using SYBR Green PCR Master Mix according to the protocol provided by the manufacturer. Relative gene expression levels were normalized by 18S rRNA or β-Actin. The primers specific for mouse GHS-R-1a were as follows: forward primer 5′-GGACCAGAACCACAAACAGACA-3′, reverse primer 5′-CAGCAGAGGATGAAAGCAACA-3′^50^, and can distinguish the functional receptor GHS-R 1a from truncated receptor GHS-R 1b.

### Glucose tolerance testing and Re-feeding experiments

To assess the effect of obestatin on insulin secretion *in vivo*, mice were fasted overnight (18 hrs) and then injected intraperitoneally (*i.p*.) with either 0.5 µmol/kg (1.26 mg/kg) obestatin^35^ or saline, and then oral glucose tolerance test (OGTT) was performed immediately by gavaging 2 g/kg glucose. Blood samples for glucose and insulin measurements were obtained at 0, 15, and 60 minutes after the administration of obestatin and glucose. For re-feeding experiments, the mice were fasted overnight (18 hrs) and were *i.p*. injected with either 0.5 µmol/kg obestatin or saline, and then provided with chow. Blood glucose and insulin samples were obtained at 0, 15, 30, 60 min after refeeding. Glucose was measured using OneTouch Ultra2 glucometer (LifeScan Inc., Milpitas, CA). Insulin in EDTA-treated plasma was measured using Mouse Insulin ELISA kit (Mercodia, Uppsala, Sweden) per the manufacturer’s instructions.

### Statistical analysis

Graph-Pad Prism version 6.0 software was used. Two-way ANOVA with repeated measures or one-way ANOVA was used for statistical analysis. Data are represented as mean ± SEM, and P < 0.05 was considered statistically significant.

## Results

### Obestatin enhances glucose-stimulated insulin secretion (GSIS)

**Figure 1:**
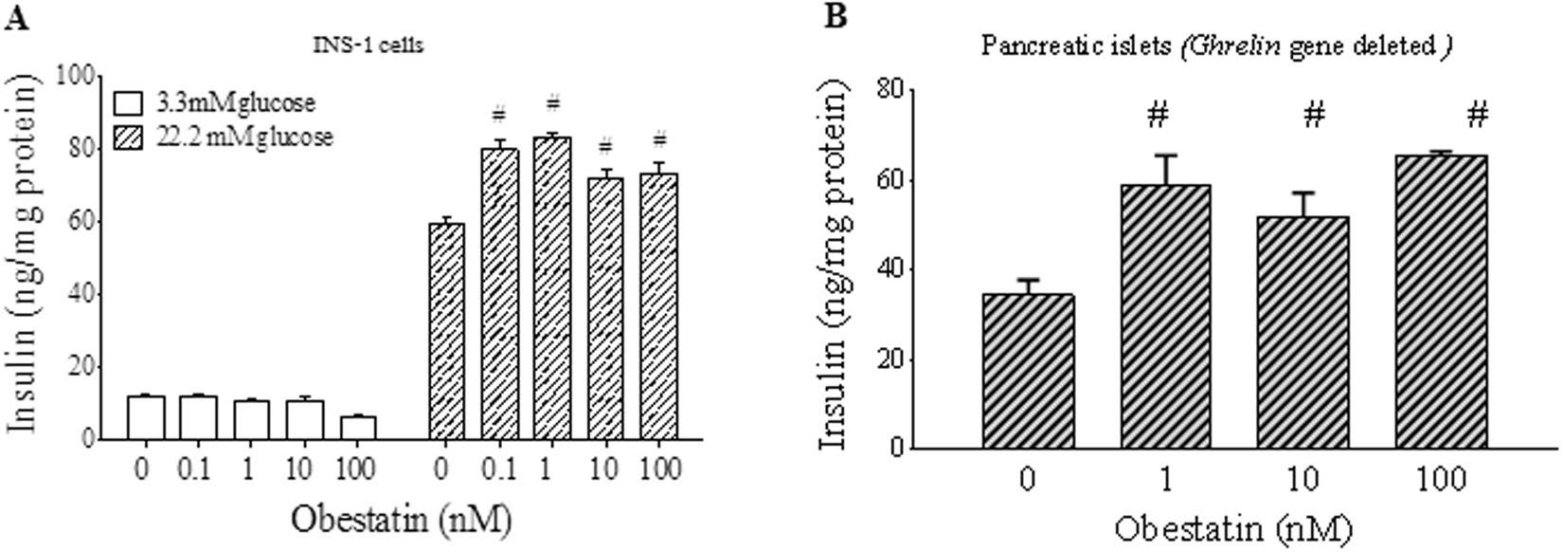
Obestatin increases glucose-stimulated insulin secretion (GSIS). β-cells or pancreatic islets were treated with different doses of obestatin in presence of 3.3 or 22.2 mM glucose for 1 hour. Insulin measured was normalized to total protein content of cells or islets. (**A**) INS-1 cells. (**B**) Pancreatic islets isolated from *ghrelin* ^−/−^ mice, and tested under glucose concentration of 22.2 mM. ^#^P < 0.05 obestatin treatment *vs* saline treatment, per glucose concentration, n = 3–5.

To determine the effect of obestatin on insulin secretion, we utilized the rat insulinoma INS-1 cells and the pancreatic islets isolated from our preproghrelin knockout mice (*ghrelin* ^−/−^) where both ghrelin and obestatin coding sequences were deleted^45^. When INS-1 cells were treated with different concentrations of obestatin, the levels of insulin secretion were similar to controls at 3.3 mM low glucose condition. However, obestatin treatment significantly increased insulin secretion compared to controls at 22.2 mM glucose hyperglycemic condition (Fig. 1A). To exclude the endogenous effects of obestatin and ghrelin, we studied obestatin-induced GSIS in pancreatic islets from *ghrelin* ^−/−^ mice. Islets were treated with obestatin at 22.2 mM glucose condition for 1 hour. Consistent with our observation in INS-1 cells, obestatin-treated islets showed significantly higher insulin secretion (Fig. 1B). Our results collectively show that exogenous obestatin treatment augments glucose-stimulated insulin secretion in β-cells and isolated pancreatic islets.

### The effect of obestatin on GSIS is mediated by GHS-R in β-cells

**Figure 2:**
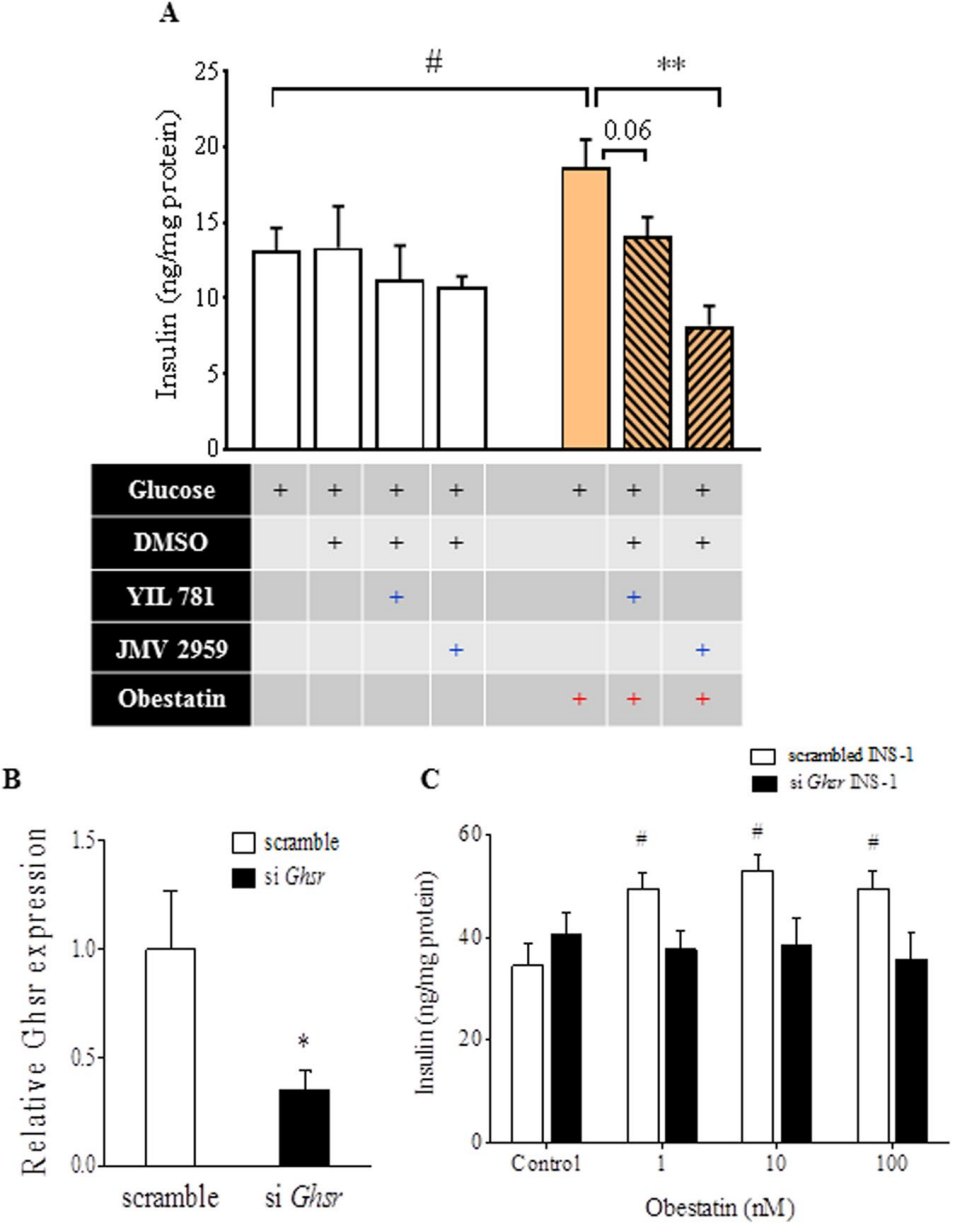
The effect of obestatin on GSIS is mediated by GHS-R in β-cells. (**A**) INS-1 cells were treated with 5 µM GHS-R antagonists YIL781 or JMV2959 for 1 hour, and insulin secretion was measured at 22.2 mM glucose with or without 100 nM obestatin. ^#^P < 0.05, treatment *vs* control; *P < 0.05 obestatin *vs* obestatin with GHS-R antagonist, n = 8–14. (**B**) GHS-R was transiently knocked down in INS-1 cells and gene expression was measured after 48 hours. (**C**) Scrambled or *siGhsr* transfected INS-1 cells were treated with different doses of obestatin at 22.2 mM glucose for 60 minutes. Insulin was normalized to total protein content of cells. ^#^P < 0.05 obestatin treatment *vs* control for each genotype, *P < 0.05 scrambled *vs siGhsr*, n = 5.

The identity of the receptor for obestatin has been elusive. In order to investigate whether the effect of obestatin on insulin secretion is mediated by GHS-R in β−cells, we pharmacologically blocked GHS-R by using GHS-R antagonists YIL781 and JMV2959 in INS-1 cells. The GHS-R antagonists alone showed no effect on insulin secretion. Treatment of 100 nM obestatin significantly increased insulin secretion from treated cells as compared to controls. However, when cells were treated with a combination of obestatin and GHS-R antagonists, the stimulatory effect of obestatin on insulin secretion was eliminated (Fig. 2A).

Next, we suppressed GHS-R expression in INS-1 cells by transiently knocking down *Ghsr* expression with siGHS-R. *Ghsr* expression in *siGhsr*-transfected cells was reduced by 60–70% as compared to scrambled siRNA treated cells after 48 hours (Fig. 2B). The scrambled and *siGhsr*-transfected cells were treated with 1 nM, 10 nM and 100 nM obestatin in the presence of 22.2 mM glucose for 1 hour. Obestatin-induced GSIS was observed with the scrambled siRNA-treated cells under different concentrations of obestatin, but not in the *siGhsr*-transfected cells (Fig. 2C). These results indicate that absence of GHS-R attenuates obestatin-induced GSIS in β-cells.

### Obestatin-induced insulin secretion is abolished in GHS-R null mice

**Figure 3:**
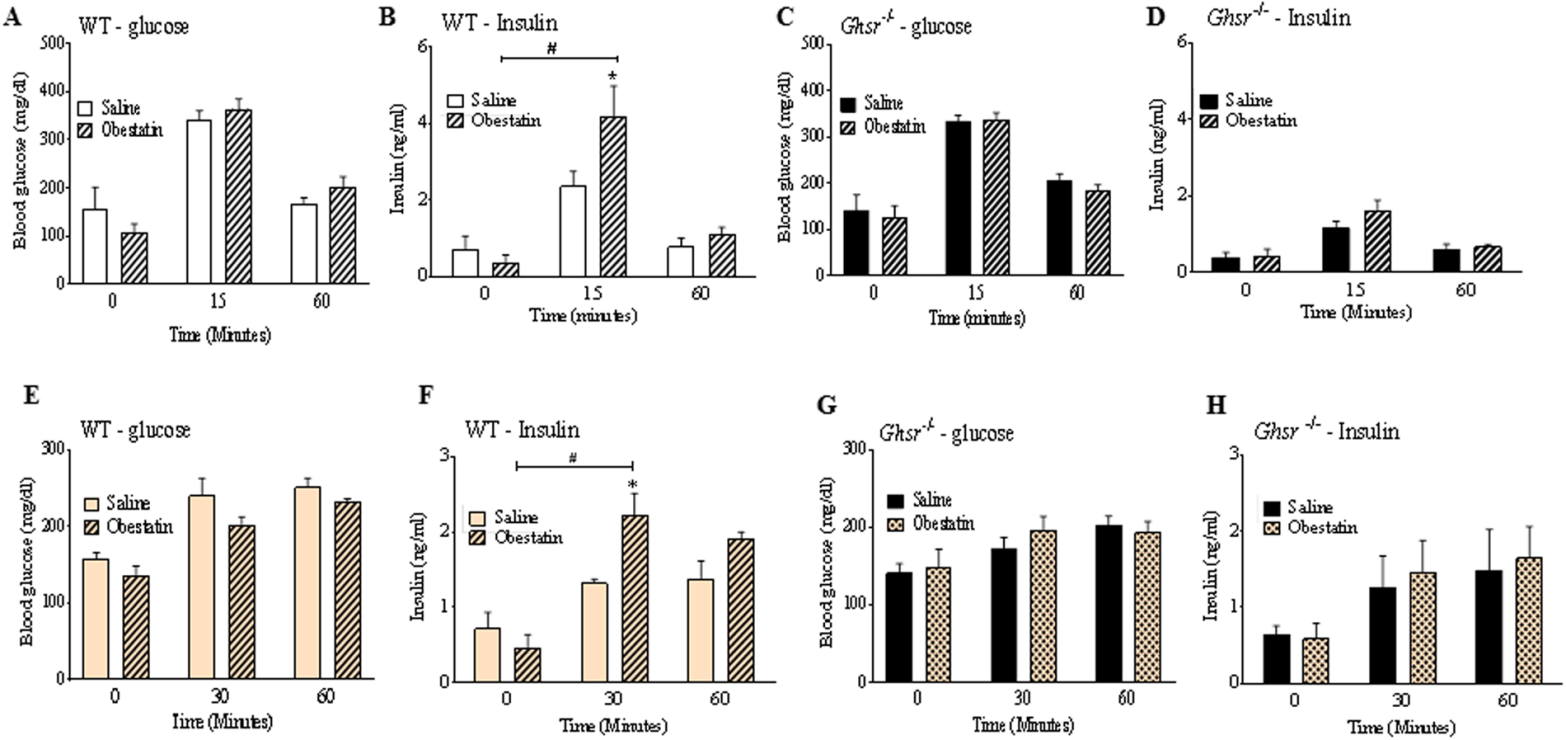
Obestatin-induced insulin secretion is abolished in GHS-R null mice. Overnight-fasted WT and *Ghsr* ^−/−^ mice were *i.p*. injected with saline or 0.5 µmol/kg obestatin, and 2 g/kg glucose was gavaged. Blood glucose in WT (**A**) & *Ghsr* ^−/−^ (**C**) and plasma insulin in WT (**B**) & *Ghsr* ^−/−^ (**D**) was measured at 0, 15 and 60 min after injection. ^#^P < 0.05 saline/obestatin treatment at 15 min or 60 min *vs* saline/obestatin treatment at 0 min, *P < 0.05 saline *vs* obestatin for each time point, n = 3–5. Overnight-fasted WT and Ghsr^−/−^ mice were fed *Ad. Lib*. chow diet after saline/obestatin injections. Blood glucose in WT (**E**) & *Ghsr* ^−/−^ (**G**) and plasma insulin in WT (**F**) and *Ghsr* ^−/−^ (**H**) was measured at 0, 30 and 60 min after injection. ^#^P < 0.05 saline/obestatin treatment at 30 min or 60 min *vs* saline/obestatin treatment at 0 min, *P < 0.05 saline *vs* obestatin for same time point, n = 6–8.

Our results in INS-1 cells show that absence of GHS-R attenuates obestatin-induced GSIS. To determine the implications of obestatin on insulin secretion *in vivo*, we further investigated obestatin-induced GSIS in *Ghsr* ^−/−^ mice. We treated overnight-fasted wild-type (WT) and *Ghsr* ^−/−^ mice with either obestatin (0.5 µmol/kg i.p) or saline, and then carried out OGTT. At 15 minutes after glucose administration: 1) obestatin-treated WT mice displayed glucose levels comparable to that of saline-treated WT mice, respectively (Fig. 3A). 2) Insulin in obestatin-treated WT mice increased from 0.37 + 0.2 ng/ml to 4.95 + 0.32 ng/ml, while insulin in saline-injected group only increased from 0.71 + 0.34 ng/ml to 2.36 + 0.38 ng/ml (Fig. 3B). Thus, obestatin-treated WT mice showed significantly higher GSIS compared to those of the saline-injected group. In contrast, glucose and insulin levels were not different between obestatin-injected *Ghsr* ^−/−^ mice and the saline-injected *Ghsr* ^−/−^ mice after 15 minutes of glucose and obestatin administration (Fig. 3C,D).

To assess the effect of obestatin on insulin secretion under physiological condition, we also investigated the effect of obestatin on insulin secretion in response to regular chow feeding. WT and *Ghsr* ^−/−^ mice were fasted overnight, then *i.p*. injected with 0.5 µmol/kg obestatin and fed with chow. After 30 minutes of feeding, glucose levels were comparable in both WT and *Ghsr* ^−/−^ mice (Fig. 3E,G). Similar to OGTT, insulin levels in obestatin-treated WT mice were significantly higher when compared to the saline-treated WT mice, with insulin levels at 2.24 + 0.28 ng/ml with obestatin and 1.33 + 0.05 ng/ml with saline at 30 minute time point (Fig. 3F). However, in *Ghsr* ^−/−^ mice, insulin levels were similar between the obestatin- and saline-treated groups at both 30 and 60 minute time points (Fig. 3H).

These data indicate that obestatin increases insulin secretion in WT mice, but this effect is absent in *Ghsr* ^−/−^ mice under both oral glucose challenge and chow feeding. Also note-worthy is that the response of obestatin-induced insulin is rapid, and this effect is diminished within an hour. These studies suggest that obestatin is an import regulator of insulin secretion *in vivo* and GHS-R is required for obestatin’s action on insulin secretion.

### Obestatin’s effect on insulin secretion is mediated directly by GHS-R in pancreatic β-cells

**Figure 4:**
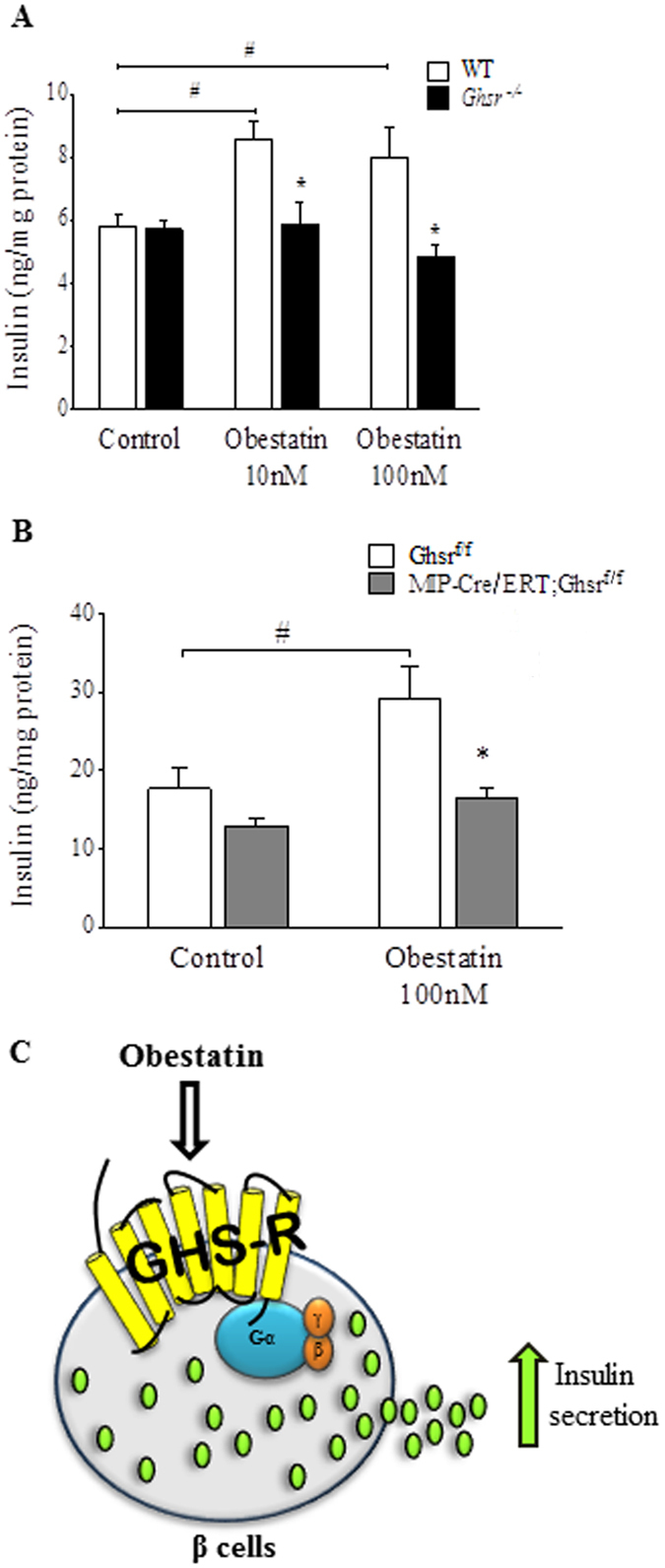
Obestatin-induced insulin secretion is mediated directly by GHS-R expression in pancreatic β-cells. (**A**) Pancreatic islets from *Ghsr* ^−/−^ mice were treated with different doses of obestatin at 22.2 mM glucose concentration for 1 hour. Insulin was normalized to total protein content of islets. ^#^P < 0.05 obestatin treatment *vs* saline control for each genotype, *P < 0.05 WT *vs Ghsr* ^−/−^ islets, n = 7–8. (**B**) Pancreatic islets from Ghsr^*f*/*f*^ and MIP-Cre/ERT;Ghsr^*f/f*^ mice were treated with 100 nM Obestatin at 22.2 mM glucose condition for 1 hour. Insulin was normalized to total protein content of islets. ^#^P < 0.05 obestatin treatment *vs* control for each genotype, *P < 0.05 Ghsr^*f/f*^ *vs* MIP-Cre/ERT;Ghsr^*f/f*^ islets for each treatment group, n = 6–8. (**C**) Schematic of obestatin augments glucose-stimulated insulin secretion via GHS-R in β-cells.

We next validated the necessity of GHS-R for obestatin-induced GSIS in pancreatic islets. We isolated pancreatic islets from WT and *Ghsr* ^−/−^ mice and treated them with obestatin at 22.2 mM glucose. Similar to our findings in the β-cells, obestatin treatment at both obestatin doses increased the insulin secretion of WT islets, but did not affect insulin secretion of *Ghsr* ^−/−^ islets (Fig. 4A). The data further demonstrated that GHS-R mediates obestatin-induced GSIS.

Since GHS-R is expressed in α, β and δ-cells of pancreatic islets, we further investigated whether GHS-R in pancreatic β-cells alone determines obestatin’s effect on GSIS. To this end, we generated β-cell-specific *Ghsr* knockout mice by breeding MIP-Cre/ERT^47^ with our Ghsr^f/f^ mice^51^. The MIP-Cre/ERT line is widely accepted as the β-cell-specific Cre line^47^. To validate the mouse model, we measured *Ghsr* expression in brain and peripheral tissues. Indeed, the reduced *Ghsr* expression was detected only in islets (around 30–34%), but not in other tissues tested (Data not shown). The reduction of *Ghsr* expression in pancreatic islets is likely due to *Ghsr* deletion in the β-cells, and the remaining GHS-R expression is likely due to the intact GHS-R expression in α and δ-cells of the islets. We isolated islets from tamoxifen-induced Ghsr^f/f^ and MIP-Cre/ERT;Ghsr^f/f^ mice, and treated them with obestatin for 1 hour at high glucose condition. Treatment of Ghsr^f/f^ control islets with 100 nM obestatin significantly increased insulin secretion compared to controls, whereas obestatin treatment of islets from MIP-Cre/ERT;Ghsr^f/f^ mice exhibited no increase of insulin (Fig. 4B). These results indicate that obestatin has direct effect on pancreatic β-cells, and that GHS-R expression in β-cells is required for obestatin-induced GSIS (Fig. 4C).

## Discussion

In the current study, we used a comprehensive set of *in vitro*, *ex vivo*, and *in vivo* systems to investigate the effect of obestatin on GSIS, and whether GHS-R mediates the effect of obestatin on insulin secretion. We have found that: 1) Obestatin augments GSIS in INS-1 β-cells and pancreatic islets; 2) Obestatin-induced GSIS is abolished by GHS-R blockade/suppression in β-cells; 3) Obestatin increases insulin secretion in WT mice, but this effect is abolished in GHS-R knockout mice; 4) Obestatin-induced GSIS is absent in pancreatic islets isolated from global and β-cell-specific GHS-R knockout mice. Taken together, our data unambiguously demonstrate that obestatin augments insulin secretion under hyperglycemic state, and the stimulatory effect of obestatin on insulin is mediated by ghrelin receptor GHS-R in pancreatic β-cells.

The effect of obestatin on insulin secretion is controversial. Qader *et al*. showed that obestatin inhibits insulin secretion in rat and mouse islets at 8.3 mM and 12 mM glucose concentrations, respectively^32^. Ren *et al*. showed that obestatin inhibits insulin secretion of pancreatic islets at high glucose concentration^7^. Egido *et al*. reported a dual, dose-dependent effect of obestatin on GSIS in perfused rat pancreas at 9 mM glucose: obestatin stimulated insulin secretion at low concentrations of obestatin (3.14 nM and 1 nM), but inhibited insulin secretion at a higher concentration of obestatin (10 nM)^3^. Several other studies have suggested that obestatin has no effect on insulin secretion^7, 33–35^.

Islets from the *ghrelin* ^−/−^ mice used in our study lack the coding sequences of both ghrelin and obestatin^45^, thus obestatin’s effect observed in *ghrelin* ^−/−^ islets is not affected by endogenous ghrelin or obestatin. The stimulatory effect of obestatin on GSIS is intact in *ghrelin* ^−/−^ mice, indicating that obestatin has direct effect on insulin secretion. In our study, injection of WT mice with 0.5 µmol/kg obestatin, and treatments of β-cells with 0.1–100 nM obestatin and pancreatic islets with 1–100 nM obestatin, all increased insulin secretion. Similar to our observations, Granata *et al*. reported that 100 nM obestatin increases insulin secretion in human islets^1^. Interestingly they found that obestatin has stimulatory effect on insulin at both high and low glucose concentrations in human islets^1^, but we only detected the effect under high glucose concentration in mouse islets. The discrepancy between high and low glucose concentrations in studies by us and Granata *et al*. could be due to difference in species of models studied. Granata *et al*. showed that the stimulatory effect of obestatin on GSIS persists under high fat diet condition^2^, and obestatin increases insulin in STZ-induced diabetic rats^26^. Recently Granata *et al*. also showed that obestatin enhances the generation of functional β cells in islet-like cell clusters derived from mouse pancreatic precursor cells, and obestatin increases insulin gene expression and C-peptide secretion from these clusters under glucose-stimulated condition^52^. These findings are well in line with our observations. The dosages of obestatin used in our study are higher than the usual physiological levels of the peptide in the blood, which range from 600 to 800 pg/ml^33^. However, the concentration of obestatin at the level of the islet is estimated to be much higher than its concentration in circulation, as obestatin is locally secreted from ε-cells in pancreatic islets^12^. It has been shown that only 70% of the circulating ghrelin originates from the stomach^53^, whereas the rest is derived from other tissues such as pancreas. The discrepancy between the reports from others and our data could be attributed to: a) species of origin of islets, b) different glycemic conditions and/or obestatin dose, c) the purity and potency of the peptide used in the studies, and/or d) duration of the obestatin treatment.

Similar to the debatable nature of obestatin’s effect on insulin secretion, the receptor mediating the actions of obestatin is even more elusive. Despite the detection of obestatin binding sites in various tissues such as pancreas and white adipose tissues^1, 41, 54^, obestatin is still considered an orphan ligand because its cognate receptor is uncertain. Originally, Zhang *et al*. suggested that obestatin’s actions may be mediated by GPR39 receptor, and demonstrated binding of obestatin to CHO cells expressing GPR39 receptor^6, 36^. Subsequent studies were not able to confirm the finding of Zhang *et al*. and failed to detect the activation of GPR39 with obestatin treatment^38–40^. Later, GLP-1R was suggested to mediate the functions of obestatin, promoting β-cell survival and proliferation^1^. However, experiments using GLP-1R overexpressing β-cells could not confirm the specific binding^35^. Interestingly, Granata *et al*. observed that besides GLP-1R, obestatin could also interact with acylated ghrelin binding sites, and obestatin-induced β-cell survival effect was ablated in the presence of GHS-R antagonist [D-Lys3]- GHRP6^1^. GHS-R is a GPCR^40^, and several studies have suggested that obestatin likely binds and activates a GPCR^41–44^. It is possible that obestatin possesses low-affinity binding for GHS-R. We demonstrated that obestatin-induced GSIS in INS-1 β-cells is dependent on GHS-R expression; obestatin-induced GSIS is abolished in *Ghsr* ^−/−^ mice, and islets isolated from *Ghsr* ^−/−^ and β-cell-specific *Ghsr* knockout mice. These results definitively demonstrate that the stimulatory effect of obestatin on insulin secretion is mediated by GHS-R in pancreatic β-cells.

GHS-R is the only recognized biologically relevant receptor of ghrelin^55^ and is expressed in the brain and peripheral tissues including intestine, endocrine pancreas, and adipose tissues^56^. We have shown that GHS-R mediates ghrelin’s effects on food intake and growth hormone secretion^18^. Dezaki *et al*. has reported that ghrelin-induced inhibition of insulin secretion in β-cells is mediated through GHS-R^57^. Obestatin and ghrelin, both products of the *preproghrelin* gene, have opposing effects on insulin secretion as demonstrated by us and others. GSIS can be potentiated or suppressed by hormones and neural substances. Extensive research has established ghrelin’s role in the regulation of insulin release and glycemic control. Ghrelin inhibits glucose-induced insulin release *in vivo*, and in perfused pancreas and isolated islets^57–59^. Our data indicate that obestatin has an insulin-stimulatory effect under hyperglycemic condition, which is opposite from the insulin-inhibitory effect of ghrelin. Several reports show that the ratio of obestatin/ghrelin is different in anorexia nervosa and obesity^60^, which suggests that the obestatin/ghrelin ratio may serve as a biomarker for different nutritional states. We have reported that fasting reduces plasma obestatin, but increases ghrelin in mice^61^. The stimulatory effect of obestatin and the inhibitory effect of ghrelin on insulin secretion are in line with their anorexic and orexigenic properties.

It is intriguing that both ghrelin and obestatin signal through GHS-R, yet have opposite effects on insulin secretion. GHS-R activation is most commonly associated with coupling of GHS-R to G protein subunit Gα_q/11_^55, 62^. Interestingly, it has been reported that GHS-R couples with Gα_i/o_ in the pancreatic islets to mediate ghrelin’s inhibitory effect on GSIS^63^. Additionally, GHS-R has been shown to form heterodimers with other GPCRs, such as dopamine receptors DRD1, DRD2^64, 65^, MC3R^66^ and 5-HT_2C_^67^ to transmit different functions. Park *et al*. showed that ghrelin inhibits insulin secretion in β cells via GHS-R1a heterodimerization with Somatostatin receptor-5 (SST5), where GHS-R1a couples to Gα_i/o_ instead of Gα_q/11_^68^. It has been reported that GHS-R1a dimerizes with D1 to switch GHS-R1a coupling from Gα_q/11_ to Gα_s_ in NPY cells of arcuate nucleus^69, 70^. We thus speculate that obestatin-induced GSIS in β-cells is mediated by the coupling of GHS-R1a to different G protein subunits: a) Gα_q/11_, to potentiate cytosolic Ca^2+^ levels subsequently increasing insulin secretion; b) Gα_s_, through heterodimerization of GHS-R with other GPCR, such as GLP-1R^71, 72^. While we have demonstrated that GHS-R is required for obestatin’s insulinotropic effect, we did not perform ligand binding assay or test response of obestatin in a heterologous GHS-R expression system. Thus we cannot definitively conclude that obestatin directly binds to GHS-R. Further studies are needed to test the direct binding of obestatin to GHS-R and/or obestatin-induced heterodimerization of GHS-R with other receptors. In depth understanding of the downstream signaling pathways mediating the opposing effects of ghrelin and obestatin in β-cells would be of interest as well.

It has been shown that obestatin plays important roles in the survival and function of pancreatic β-cells. Obestatin treatment improves the survival of β-cells and human islets by reducing apoptosis^1^. Obestatin enhances insulin sensing of pancreatic islets and streptozotocin-induced β-cells^2, 32^. Insulin receptor substrate 2 (IRS-2) was reported to reduce the incidence of diabetes in IRS-2-overexpressing non-obese diabetic mice^73^. It has been suggested that obestatin improves insulin sensitivity by increasing expression and phosphorylation of IRS-2^1^. Our comprehensive *in vitro*, *ex vivo and in vivo* dataset collectively demonstrate that obestatin is also a potent insulin secretagogue, and the stimulatory effect of obestatin on insulin secretion is mediated by ghrelin receptor GHS-R in β-cells. Our finding of obestatin’s stimulatory effect on insulin combined with the other beneficial effects of obestatin on β-cells, makes obestatin an extremely attractive multifunctional therapeutic candidate for Type 2 diabetes.
